# Bacteriophage Cocktail and Microcin-Producing Probiotic *Escherichia coli* Protect Mice Against Gut Colonization With Multidrug-Resistant *Escherichia coli* Sequence Type 131

**DOI:** 10.3389/fmicb.2022.887799

**Published:** 2022-04-25

**Authors:** Stephen B. Porter, Brian D. Johnston, Dagmara Kisiela, Connie Clabots, Evgeni V. Sokurenko, James R. Johnson

**Affiliations:** ^1^Minneapolis VA Health Care System, Veterans Health Administration, United States Department of Veterans Affairs, Minneapolis, MN, United States; ^2^Department of Medicine, University of Minnesota, Minneapolis, MN, United States; ^3^Department of Microbiology, University of Washington, Seattle, WA, United States

**Keywords:** *Escherichia coli*, intestinal colonization, bacteriophage, probiotic, multidrug resistance, microcin, mouse model, ST131

## Abstract

Non-antibiotic measures are needed to reduce the rate of infections due to multidrug-resistant organisms (MDROs), including by eliminating the commensal reservoir that underlies such strains’ dissemination and leads to recurrent infections. Here, we tested a cocktail of pre-selected bacteriophages and an engineered microcin C7-producing probiotic *Escherichia coli* Nissle-1917 strain for their ability to reduce gut colonization by an *E. coli* strain from sequence type 131 (ST131)-*H*30R, which is the major clonal group of MDROs among extraintestinal clinical *E. coli* isolates. Although the bacteriophage cocktail was highly effective against ST131-*H*30R strains both *in vitro* and in a murine model of subcutaneous sepsis, it was only weakly and transiently effective against gut colonization by the target ST131-*H*30R strain (0.5 log_10_ decrease on *d* + 1: *p* < 0.001; no significant effect on *d* + 4 and beyond). The probiotic strain, while also highly active against ST131-*H*30R *in vitro*, was ineffective against ST131-*H*30R gut colonization despite its abundant presence in feces. Nonetheless, despite failing as decolonizing agents when administered separately, when co-administered the bacteriophage cocktail and probiotic strain exhibited striking synergy against ST131-*H*30R gut colonization. This combinatory effect was most pronounced on *d* + 1 (3.3 log_10_ target strain decrease: *p* < 0.001), and persisted until *d* + 7 (0.5 log_10_ decrease; *p* < 0.02.). Although by *d* + 10 the ST131-*H*30R load was fully restored, these findings provide proof of concept for combined bacteriophage-plus-probiotic administration to reduce or, possibly, to prevent gut colonization with MDROs in high-risk individuals.

## Introduction

*Escherichia coli* sequence type 131 (ST131), and specifically its pandemic fluoroquinolone-resistant *H*30R subset, has become the single most common cause of extraintestinal *E. coli* infections, and is now responsible for two-thirds or more of multidrug-resistant *E. coli* infections (Nicolas-Chanoine et al., 2014; Johnson et al., 2017). *H*30R comprises two main sister subclones: *H*30Rx, which is associated with the CTX-M-15 extended-spectrum beta-lactamase (ESBL), and *H*30R1, which historically was cephalosporin-susceptible but now is an important vehicle for the CTX-M-14 and CTX-M-27 ESBLs (Price et al., 2013; Petty et al., 2014; Johnson et al., 2016; Matsumura et al., 2016; Stoesser et al., 2016).

The emergence and dissemination of ST131-*H*30R may relate to its known exceptional persistence in humans as a gut colonizer, which may increase its opportunity to cause extraintestinal infections and to spread among hosts (Johnson et al., 2016, 2022; Overdevest et al., 2016; Tchesnokova et al., 2020). As such, measures to eliminate or block gut colonization with *H*30R conceivably could help in preventing infections in high-risk individuals by breaking the cycle of recurrent infections, thereby combatting the MDRO pandemic in general.

Because of the already-extensive antimicrobial resistance of *H*30R and the risk of selecting for additional resistance, non-antibiotic measures are preferable to antibiotics as anti-colonization measures. Two promising non-antibiotic modalities are bacteriophages (i.e., nontoxic antibacterial viruses; Loc-Carillo and Abedon, 2011; Lin et al., 2017; Weber-Dabrowska et al., 2017; El Haddad et al., 2019) and probiotics (i.e., nonpathogenic antibacterial bacteria; Paton et al., 2006; Dobson et al., 2012; Amalaradjou and Bhunia, 2013; Fijan, 2014).

Bacteriophage has shown promise for treating diverse experimental *H*30R infections, as induced in rodents either by direct inoculation (Wang et al., 2006; Pouillot et al., 2012; Dufour et al., 2016; Green et al., 2017) or by spontaneous translocation of gut-resident bacteria after cyclophosphamide treatment (Green et al., 2017). By contrast, for bacteriophage-mediated gut decolonization, results to date have been inconsistent with non-ST131 organisms (Waseh et al., 2010; Galtier et al., 2016; Contreras et al., 2019; Javaudin et al., 2021a,b), and with *H*30R the only available data are from a long-term (fermenter) culture system designed to mimic the gut milieu (Bernasconi et al., 2020). Regardless of context, development of bacteriophage resistance is a known Achilles heel of bacteriophage therapy, which use of phage cocktails (Loc-Carillo and Abedon, 2011; Weber-Dabrowska et al., 2017) and/or adjunctive agents (Amankwah et al., 2021) may help to overcome or forestall.

Probiotics can decrease pathogen abundance in the gut (Piewngam et al., 2021). However, *E. coli* Nissle-1917 (hereafter, EcN), a nonpathogenic commensal strain with a long history of probiotic use in humans (Hancock et al., 2010), was ineffective in pigs against gut colonization with ESBL-producing *Enterobacteriaciae* (Mourand et al., 2017). Likewise, a mixture of eight probiotic strains was similarly inefficacious in humans (Ljungquist et al., 2020).

Notably, antimicrobial peptides (bacteriocins), as produced by probiotic bacteria, can synergize with bacteriophage to eradicate biofilms (Amankwah et al., 2021), which typify the intestinal milieu (Motta et al., 2021). Thus, combined probiotic plus bacteriophage therapy may be relevant for intestinal decolonization. To enhance their effect on target organisms, probiotics can be engineered to overexpress small bacteriocins (microcins; Forkus et al., 2017). For example, microcin C7 (Mcc7), the smallest microcin known (a heptapeptide), is produced by *E. coli* strains that harbor a plasmid-borne cassette containing the *MccABCDEF* gene cluster, which encodes Mcc7 synthesis, excretion, and immunity (Severinov and Nair, 2012). Mcc7 undergoes extensive post-translational modification, resulting ultimately in a toxic aspartyl-adenylate analogue that inhibits target-cell aspartyl-tRNA synthetases (Zukher et al., 2014).

In this study, we assessed both a novel bacteriophage cocktail and a novel microcin C7-producing derivative of probiotic strain EcN, singly and in combination, to reduce colonization of mice with a CTX-M-15-producing *H*30Rx strain.

## Materials and Methods

### *Escherichia coli* Strains

Wild-type ST131-*H*30R challenge strains for the mouse models were selected from ST131 subclones *H*30R1 (strains MVAST36 and MVAST392) and *H*30Rx (strains MVAST412, JJ2050, and JJ1886; Tables 1, 2). These strains, which exhibited O:H type O25b:H4, were selected in part based on the identification of bacteriophages active against them, as described below (Table 2), and for phylogenomic diversity within the *H*30R lineage (Price et al., 2013). Control strains for the murine sepsis model included UTI isolate CFT073 (positive control; Mobley et al., 1990a; Welch et al., 2002) and laboratory strain MG1655 (negative control; Welch et al., 2002).

**Table 1:** *Escherichia coli* strains used.

Strain name	Description	Study role	Source	References
EcN	*E. coli* Nissle 1917; human probiotic (Europe); ST73, phylogroup B2	Parent for EcN-SR; phage screen	S. Weissman	Hancock et al., 2010
EcN-SR	Spontaneous STM- and RIF-resistant mutant of EcN	Recipient for pp70 (microcin C7-encoding plasmid)	This study	n.a.
EcN-SR/pp70	EcN-SR transformed with microcin C7-encoding plasmid pp70	Microcin C7-producing derivative of EcN; used in gut model	This study	n.a.
JJ1886	ST131-*H*30Rx clinical isolate (urosepsis, cystitis); CTX-M-15	Sepsis model; JJ1886-S parent; phage harvest (sewage) and screen	Clinical	Owens et al., 2011; Price et al., 2013
JJ1886-S	Spontaneous STR-resistant mutant of JJ1886; RIF-susceptible	Used in gut model as target strain	This study	n.a.
MG1655	Laboratory *E. coli* strain; ST10, phylogroup A	Phage harvest (sewage); sepsis model neg. control (no lethality)	C. Dozois	Welch et al., 2002
CFT073	Clinical isolate (pyelonephritis); ST73, phylogroup B2	Sepsis model pos. Control (lethal to four or five of five mice)	H. Mobley	Mobley et al., 1990b; Welch et al., 2002
MVAST36	ST131-*H*30R1	Sepsis model; phage harvest (sewage) and screen	Clinical	Price et al., 2013
JJ2050	ST131-*H*30Rx; CTX-M group 9	Sepsis model; phage harvest (sewage) and screen	Clinical	Johnson et al., 2012
MVAST392	ST131-*H*30R1	Sepsis model; phage screen	Clinical	Johnson et al., 2012
MVAST412	ST131-*H*30Rx; ESBL-negative	Sepsis model; phage screen	Clinical	Tchesnokova et al., 2018
JJ2528	ST131-*H*30Rx; ESBL non-CTX-M-15, non-CTX-M group 9	Phage harvest (sewage) and screen	Clinical	Johnson et al., 2012
JJ2555	ST131-*H*30Rx; CTX-M-15	Phage screen	Clinical	Johnson et al., 2012
CU758	ST131-*H*30R1	Phage harvest (sewage) and screen	Clinical	Johnson et al., 2010
MVAST46	ST131-*H*30R1	Phage screen	Clinical	Price et al., 2013
MVAST84	ST131-*H*30R1	Phage screen	Clinical	Price et al., 2013
JJ2134	ST131-*H*30Rx; ESBL-negative	Phage screen	Clinical	Johnson et al., 2012
JJ2183	ST131-*H*30Rx; ESBL-negative	Phage screen	Clinical	Johnson et al., 2012
ED1a	Probiotic strain; ST452, phylogroup B2	Phage screen	E. Denamur	Mourand et al., 2017
ECOR20	ST48, phylogroup A	Phage screen	H. Ochman	Ochman and Selander, 1984
ECOR48	ST70, phylogroup D	Phage screen	H. Ochman	Ochman and Selander, 1984
ECOR58	ST75, phylogroup B1	Phage screen	H. Ochman	Ochman and Selander, 1984
ECOR62	ST79, phylogroup B2	Phage screen	H. Ochman	Ochman and Selander, 1984
ECOR31	ST57, phylogroup E	Phage screen	H. Ochman	Ochman and Selander, 1984
ECOR36	ST60, phylogroup F	Phage screen	H. Ochman	Ochman and Selander, 1984
ECOR70	ST88 (STc23), phylogroup C	Phage screen	H. Ochman	Ochman and Selander, 1984

*EcN, *E. coli* Nissle 1917; ECOR, *E. coli* reference collection; ESBL, extended-spectrum beta-lactamase; Gp9, group 9 CTX-M ESBL; neg., negative (negative lethality control or no detected ESBL gene); pos., positive (positive lethality control); R, rifampin-resistant (spontaneous mutant); RIF, rifampin; S, streptomycin-resistant (spontaneous mutant); ST, sequence type; STc, ST complex (i.e., group of closely related STs); and STM, streptomycin*.

**Table 2:** Spectrum of activity of 10 *Escherichia coli*-lytic bacteriophages recovered from municipal sewage from Seattle, WA and St. Paul, MN, United States.

Bacteriophage activity^a^ against indicated *E. coli* target strain																								
Phage^b^	Target strain used for initial phage enrichment from sewage	Selected for use in phage cocktail	ST131-*H*30R												Non-ST131								
			**MVAST 36 (ST131-*H*30R1)**	**MVAST 392 (ST131-*H*30R1)**	**JJ1886**^**c**^ **(ST131-*H*30Rx, CTX-M -15)**	**JJ2050 (ST131-*H*30Rx, CTX-M Gp9)**	**MVAST 412 (ST131-*H*30Rx, no ESBL)**	CU758 (ST131-*H*30R1)	MVAST 46 (ST131-*H*30R1)	MVAST 84 (ST131-*H*30R1)	JJ2528 (ST131-*H*30R1, ESBL-other)	JJ2555 (ST131-*H*30Rx, CTX-M -15)	JJ2134 (ST131-*H*30Rx, ESBL-neg.)	JJ2183 (ST131-*H*30Rx, ESBL-neg.)	Nissle 1917 (ST73, phylo group B2)	ED1a (ST452, phylo group B2)	ECOR 20 (ST48, phylo group A)	ECOR 48 (ST70, phylo group D)	ECOR58 (ST75, phylo group B1)	ECOR62 (ST79, phylo group B2)	ECOR31 (ST57, phylo group E)	ECOR36, (ST60, phylo group F)	ECOR70 (ST88 [STc23], phylo group C)	Comment
MV36.2	MVAST36	Yes	3 s	0	0	0	1	0	0	0	0	0	0	0	0	0	0	0	0	0	0	0	0	Strong ST131 activity only with MVAST36
JJ2050.2	JJ2050	Yes	3 s	0	3	3	3	0	0	0	0	0	0	0	0	0	0	0	0	0	0	0	0	*H*30Rx and *H*30R1 activity
C3	JJ1886	Yes	0	0	3	3	3	0	0	0	0	0	0	0	0	0	0	0	0	0	0	0	0	*H*30Rx activity only
C19T	JJ1886	Yes	3	3	3 s	3	1	1	0	1	2	1	2	0	0	0	0	0	0	0	0	0	0	Broad ST131 activity
JJ6.1	JJ1886	Yes	0	0	3	3	3	0	0	0	0	0	0	0	0	0	0	0	0	0	0	0	0	Same profile as phage C3; different source
MV36.7a	MVAST36	No	3 s	0	2	2	1	3 s	0	0	2	2	3	0	0	0	0	0	0	0	0	0	0	After purification, duplicated MV36.2
JJ2528.3	JJ2528	No	0	0	0	0	0	3 s	0	0	3	0	0	0	3	0	0	0	0	0	0	0	0	Lyses Nissle 1917; exclude
C12	MG1655	No	3	0	1	1	0	1	0	0	0	2	0	0	0	0	0	0	0	0	0	0	0	Strong ST131 activity only with MVAST36
JJ6.2	JJ1886	No	0	0	3	3	0	0	0	0	0	0	0	0	0	0	0	0	0	0	0	0	0	Limited spectrum
CU758.1	CU758	No	0	0	0	0	0	3	0	0	3	0	0	0	3	0	0	0	3 s	0	0	0	0	Lyses Nissle 1917 and ECOR58; exclude
Cocktail^d^	Multiple^e^	NA	3	3	3^c^	3	3	nd	nd	nd	nd	nd	nd	nd	nd	nd	nd	nd	nd	nd	nd	nd	nd	

*Boldface: strains selected for administration to mice in the sepsis model*.

*ESBL, extended-spectrum beta-lactamase; Gp9, group 9 CTX-M; nd, not done; NA, not applicable; ST, sequence type; and STc, ST complex*.

a *Bacteriophage activity scored as 0–3, where 0 = no discernible inhibition or lysis; 1 = faint inhibition at inoculation point; 2 = lytic zone with abundant internal colonies; 3 s = lytic zone with few, small internal colonies; and 3 = lytic zone with no internal colonies*.

b *Phages C3, C12, and C19T were from Seattle, WA, United States; all others were from St. Paul, MN, United States. Phages JJ6.1, JJ6.2, and CU758.1 were from an initial St. Paul sewage sample; phages MV36.2, MV36.2a, JJ2050.2, and JJ2528.3 were from subsequent St. Paul sewage sample*.

c *The final pentavalent bacteriophage cocktail exhibited similar potency against JJ1886-S (used in the gut model) as against JJ1886, i.e., 3+ lysis*.

d *The final pentavalent bacteriophage cocktail contained bacteriophages MV36.2, JJ2050.2, C3, C19T, and JJ6.1*.

e **H*30R strains used (separately) to isolate the five selected bacteriophages included MVAST36 (phage MV36.2), JJ1886 (phages C3, C19T, and J6.1), and JJ2050 (phage JJ2050.2)*.

For the intestinal colonization model, spontaneous streptomycin-resistant mutants of parent strains JJ1886 (ST131-*H*30Rx, which caused fatal urosepsis in the source patient; Owens et al., 2011) and EcN were selected by plating dense bacterial suspensions on streptomycin-containing agar (30 mg/L; Table 1). Colonies from these plates were re-purified, confirmed molecularly as corresponding clonally with the parent (Berg et al., 1994), and designated as JJ1886-S and EcN-S. A spontaneous rifampin-resistant mutant of EcN-S was derived similarly by using rifampin-containing agar (50 mg/L), confirmed molecularly as corresponding with the parent, and designated EcN-SR.

A preliminary screen of microcins C7, J25, L, N, and V for activity against ST131-*H*30R strains showed that microcin C7 was most broadly and potently active (not shown). Consequently, to create a microcin C7-producing derivative of EcN-SR we transformed EcN-SR with plasmid pp70 (generously provided by Dr. Konstantin Severinov, Rutger’s Wacksman Institute; Zukher et al., 2014). pp70 contains a microcin C7 expression/secretion/immunity cassette in a pBR322 backbone, and encodes ampicillin resistance (Table 1). Filter-sterilized supernatants of the transformant, which was designated EcN-SR/pp70, were confirmed as exhibiting potent *in vitro* inhibition (typically, by 5–6 log_10_ CFU) of diverse ST131-*H*30R strains when combined with the target strains during log-phase growth, as described in a subsequent section.

A panel of diverse *E. coli* target strains (Tables 1, 2) was used to assess the spectrum of activity of presumptive bacteriophage-containing solutions, which was done as described below. These target strains included 12 ST131 strains [six *H*30R1 and six *H*30Rx; four of these were ESBL-positive (2 CTX-M-15, 2 CTX-M non-15)], plus representatives for three other STs within group B2, and one representative each for phylogroups A, B1, C, D, E, and F (Table 1).

### Microcin Methods

To generate solutions containing microcin C7, EcN-SR/pp70 was incubated overnight at 37°C in static, non-antibiotic-supplemented LB broth, which was then centrifuged and filter-sterilized (0.45 micron filter). EcN-SR without pp70 was used in parallel as a no-microcin control. For activity testing, a 1:1 mixture of the sterilized supernatant and fresh LB broth was distributed into 24-well tray (900 μl per well). Separately, relevant target strains (e.g., the JJ1886-S control, or JJ1886-S isolates from mouse fecal samples, as described below) were grown at 37°C in LB broth to exponential phase. After turbidity adjustment of the broths to McFarland 1.0 (~10^7^ cfu/ml), 10X serial dilutions in PBS were used to inoculate microcin-containing trays (100 μl per well). After overnight incubation at 37C, wells were scored for growth/no growth. The limiting bacterial dilution that yielded growth was compared between test and control target strains (to assess different test strains’ susceptibility to microcin C7), or between microcin-containing and control wells (to assess the microcin-producing ability of different EcN-SR/pp70 isolates).

### Phage Methods

Bacteriophages with activity against ST131-*H*30Rx were recovered from influent sewage newly obtained from municipal sewage facilities in St. Paul, MN, and Seattle, WA, United States. Raw sewage was centrifuged (2 min, 3,000 rpm) to pellet solids. Portions of the supernatant were filter-sterilized (0.45 micron filter), then spotted (10 μl) in 10x serial dilutions in PBS onto a series of Mueller-Hinton (MH) agar plates that had been overlain with 3 ml soft agar (7.5 gm/L) containing 10^6^ CFU/ml of a target *E. coli* strain (ST131-*H*30R: JJ1886, MVAST36, JJ2050, JJ228, and CU758; non-ST131: MG1655; Table 1).

After overnight incubation at 37°C, all plates showed complete lysis of the bacterial lawn within the supernatant-spotted areas out to the 10^−2^ dilution; hazy growth or isolated breakthrough colonies at intermediate dilutions; and little or no lysis by the 10^−7^ and higher dilutions. From each plate containing an ST131-*H*30R target strain, the agar beneath the undiluted supernatant plaque was excised and incubated overnight with the cognate target strain at 37°C in Luria-Bertani (LB) broth. The following day the broth was centrifuged, filter-sterilized, and spread in serial 10x dilutions in PBS onto a series of MH or LB agar plates that had been overlain with soft agar containing 10^6^ CFU/ml of the cognate target strain.

After incubation overnight at 37°C, for each target strain, plaques were harvested (as described above) from the plate with the best-separated plaques, giving priority to different-appearing plaques, which were then processed in the same manner as the initial (sewage-supernatant-derived) plaques. Three rounds of such plaque purification, each involving serial dilutions from a single plaque, were done to obtain presumably pure bacteriophage preparations.

Bacteriophage preparations were assessed for spectrum of activity by spotting them individually onto a series of MH agar plates, each overlain with a different target *E. coli* strain (Table 2). Bacteriophages were presumed to be distinct if they exhibited a unique spectrum of activity or were from different sources.

For combined use in the mouse experiments, five distinct bacteriophages were selected based on potency against ST131-*H*30R, number of ST131-*H*30R target strains lysed, and lack of activity against non-ST131-*H*30R strains, especially EcN. Individually, the selected bacteriophages exhibited 3+ activity against from 1 to 4 (median, three) of the five target strains to be used in the sepsis model; conversely, each of the five target strains was lysed (3+ level) by from 1 to 4 (median, three) of the five selected bacteriophages (Table 2). Accordingly, the resulting pentavalent cocktail exhibited 3+ activity against each of the five mouse-model target strains. The five individual bacteriophages and the pentavalent cocktail were similarly active against JJ1886-S (used in the gut model) as against the JJ1886 parent (Table 2).

For administration to mice, preparations of individual phages were generated by inoculating appropriate volumes of LB broth (usually, 20 ml) with a purified bacteriophage preparation (usually, 50 μl) and the cognate ST131-*H*30R strain (Table 2), incubating this mixture overnight with shaking at 37°C, pelleting the solids, and filter-sterilizing the supernatant. Equal volumes of each individual phage preparation were combined to give the final five-phage cocktail. Phage titer was checked by spotting serial 10X dilutions of this preparation onto target-strain-overlain LB top agar plates, as described above.

### Ethics Approval

The MVAHCS Institutional Animal Care and Use Committee (IACUC) approved the animal experimentation protocols.

### Sepsis Model

Using an established murine subcutaneous sepsis model (Merino et al., 2020), female Swiss Webster mice (Envigo ND4; 7 weeks old; mean weight 23 g; and 10 mice for each of the 10 study arms) were inoculated subcutaneously with 100 μl of a suspension of one of the five ST131-*H*30R challenge strains (total dose: 1.3–2.0 × 10^8^ CFU; log-phase growth), which immediately (≤30 min) before inoculation was combined with 100 μl of either PBS (controls) or the bacteriophage cocktail [10^7^–10^9^ plaque-forming units (PFU)/ml; total dose, 10^6^–10^8^ PFU], as prepared within 48 h of use and stored at 4°C. In parallel, five mice each were challenged with reference strains CFT073 (positive control: lethal to four or five of five mice) and MG1655 (negative control: no observable mouse illness; Table 1), and with the bacteriophage cocktail alone (negative control: no observable mouse illness whatsoever). To minimize potential artifacts from cohort or temporal effects, mice from a given shipment were allocated randomly to the different treatment arms, which were run in parallel within each experiment.

Following inoculation on d0, mice were assessed for illness severity daily for 3 days (AM and PM of *d* + 1 and *d* + 2; AM of *d* + 3), after which any surviving mice were euthanized. An experienced observer rated illness severity on a five-point scale (0, healthy; 1, mildly ill; 2, moderately ill; 3, severely ill; and 4, dead) according to standardized criteria (Merino-Velasco et al., 2017). Mice that reached stage 3 were euthanized and scored as stage 4 (dead) for any subsequent time points. Severity scores for a given mouse were averaged across the five time points. To confirm specificity, post-mortem spleen cultures were done for the two sickest mice per challenge strain. PCR-based genomic profiles (Berg et al., 1994) of *E. coli* colonies from spleen cultures uniformly corresponded with the inoculated strain (not shown).

### Intestinal Colonization Model

An established streptomycin-treated mouse intestinal colonization model was used (Lescat et al., 2017). Female Swiss-Webster mice (Envigo ND4; 5–10 weeks old; mean weight 23.5 g; and 16 per study arm) were housed individually to avoid cross-contamination. Mice, randomly assigned within each mouse shipment to four parallel treatment groups (*n* = 16 each; divided as 4 per week × 4 weeks), began continuous streptomycin treatment *via* the drinking water (5 g/L) at *d*−3 before bacterial challenge, to eliminate endogenous aerobic gut bacteria, as confirmed by fecal cultures. They then underwent oral gavage with various test substances on d0, *d* + 3, and + *d*5.

Specifically, to establish colonization with JJ1886-S, on *d*0 all mice received by gavage *H*30Rx target strain JJ1886-S (mean dose, 2.3 × 10^8^ CFU). As potential decolonization interventions, on all three gavage days (*d*0, *d* + 3, and +*d*5) mice received by gavage one of four treatments: (i) PBS (negative control), (ii) bacteriophage alone (dose, 10^6^–10^8^ PFU), (iii) EcN-SR/pp70 alone (mean dose, 2.4 × 10^8^ CFU), or (iv) bacteriophage plus EcN-SR/pp70 (each dosed as when given alone). On *d*0, the decolonization intervention dose was co-administered with target strain JJ1886-S, immediately (≤ 30 min) after these suspensions were combined. Additionally, for continuous probiotic exposure, for mice assigned to receive EcN-SR/pp70 either alone or with bacteriophage, EcN-SR/pp70 was added to the drinking water (final concentration, 10^8^ CFU/ml), which was replenished on *d*0, *d* + 3, and + *d*5 (In pilot experiments, after 48 h in ambient-temperature water EcN-SR/pp70 exhibited 80% viability and remained ampicillin-resistant).

Fresh fecal pellets were collected from mice on days −3, 0, +1, +4, +7, and +10 by moving mice to a clean, solid-bottom cage until they defecated. Pellets were weighed and dispersed in PBS, which was then cultured quantitatively on agar plates supplemented, separately, with streptomycin (30 mg/L; selects for both JJ1886-S and EcN-SR/pp70), ciprofloxacin (4 mg/L; selects for JJ1886-S), or rifampin (50 mg/L; selects for EcN-SR/pp70). If the estimated concentrations of JJ1886-S and EcN-SR/pp70 differed by <10X, for greater precision 20 colonies from streptomycin-supplemented plates were replica-plated to both ciprofloxacin-supplemented and rifampin-supplemented plates, and the observed ratio or its inverse was applied to the total cell count from streptomycin-supplemented plates. Additionally, selected colonies of putative EcN-SR/pp70 were replica-plated to ampicillin-containing plates (50 mg/L) to confirm plasmid retention. For selected isolates from each study group, strain identity was confirmed by PCR-based genomic profiling.

### Explanatory Testing of Post-challenge Isolates and Fecal Specimens

Additional experiments were done to clarify selected aspects of the intestinal colonization model results. First, to confirm the presence and functionality of bacteriophage in the mouse gut, filter-sterilized supernatants from homogenized fecal pellets from bacteriophage-treated and concurrent control mice were tested (as described above) for lytic activity against the input JJ1886-R strain. Second, to confirm sustained microcin production by EcN-SR/pp70 after residence in the mouse gut, supernatants from broth-grown fecal isolates of EcN-SR/pp70 from mice treated with EcN-SR/pp70 were tested for inhibitory activity against the input JJ1886-S strain. Third, to assess for possible development of bacteriophage and/or microcin C7 resistance by JJ1886-S during gut residence, fecal isolates of JJ1886-S from bacteriophage-treated mice were tested (as described above) for susceptibility to the input bacteriophage cocktail; likewise, fecal isolates of JJ1886-S from EcN-SR/pp70-treated mice were tested (as described above) for inhibition by supernatants from the broth-grown input EcN-SR/pp70 strain.

### Statistical Methods

For bacterial counts, between-group comparisons were tested using unpaired *t*-tests, assuming equal variance, and within-mouse comparisons between different strains were tested using paired *t*-tests. For mortality, between-group comparisons of proportions were tested using Fisher’s exact test. All *p* values were two-tailed. The significance criterion was *p* < 0.05.

## Results

### Bacteriophage Effect in the Sepsis Model

**Figure 1:**
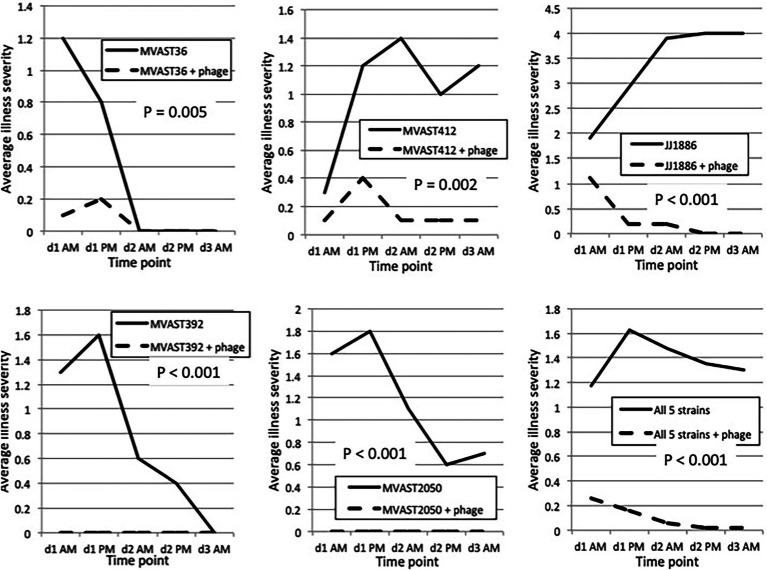
Murine sepsis model time trends. Mice were inoculated subcutaneously on *d*0 with the indicated *H*30R challenge strain combined with either PBS control or bacteriophage cocktail (“+ phage”), then assessed for illness severity (range, 0–4) at five subsequent time points (twice on *d*1 and *d*2, once on *d*3). Data shown are the average illness severity values at each time point for the 10 mice per group. *p* values (by two-tailed unpaired *t*-tests) are for between-group comparisons involving average illness severity per mouse across the five time points.

**Figure 2:**
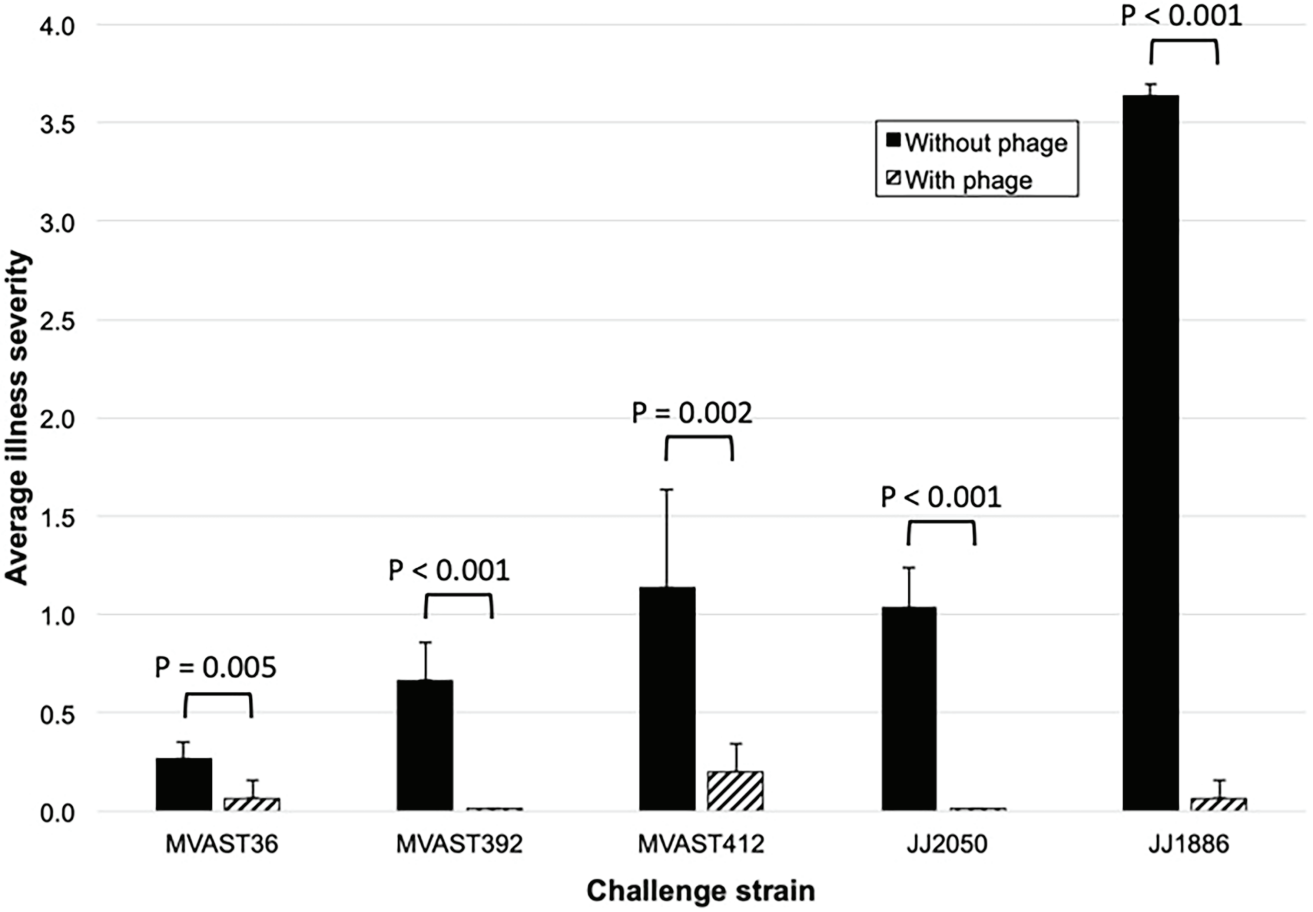
Murine sepsis model summary data. Data shown represent the average illness severity (range, 0–4) for all 10 mice per group across the five observation points (Figure 1). *p*-values (by two-tailed unpaired *t*-tests) are for between-group comparisons involving average illness severity per mouse across the five time points.

To test whether the bacteriophage cocktail is active *in vivo* against various ST131-*H*30R strains, we used the murine subcutaneous sepsis model and five ST131-*H*30R test strains (two *H*30R1, three *H*30Rx). The bacteriophage cocktail by itself caused no sign of illness in control mice, which remained as healthy throughout as did the PBS controls (*n* = 5). When inoculated without bacteriophage, the test bacteria caused distinct patterns of disease, ranging from transient mild-to-moderate illness by H30R1 strains (MVAST36 and MVAST392), through sustained more severe illness (MVAST412, JJ2050) or rapidly progressive lethal sepsis (JJ1886) by the *H*30Rx bacteria (Figure 1). Results for a given strain were highly consistent across the corresponding 10 mice (not shown). For each strain the addition of bacteriophage cocktail to the inoculum immediately before inoculation significantly attenuated disease severity, as assessed for all five strains by the average illness severity score (Figures 1, 2) and, for strain JJ1886, by percent dead (0/10 with phage, vs. 10/10 without phage: *p* < 0.001).

### Bacteriophage and Probiotic Effects in the Intestinal Colonization Model

**Figure 3:**
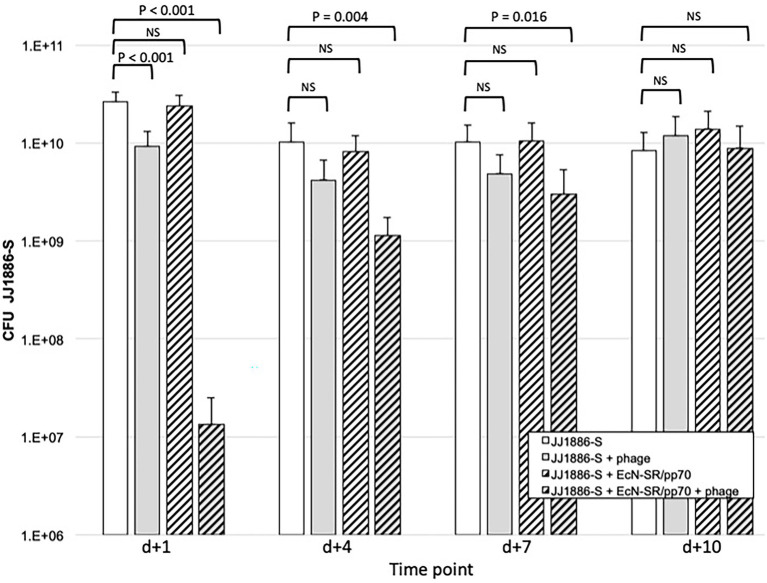
Fecal counts of strain JJ1886-S in the murine gut colonization model. Data are group mean fecal counts of JJ1886-S (*n* = 16 mice per group) at four time points post administration (*d*0) of JJ1886-S, plus one of four treatments: EcN-SR/pp70, the five-bacteriophage cocktail (“+ phage”), both, or neither. Dosing with bacteriophage and/or EcN-SR/pp70 was continued through *d* + 5 (bacteriophage) or *d* + 7 (EcN-SR/pp70). *p* values are from two-tailed unpaired *t*-tests. NS, not significant (*p* ≥ 0.05). Error bars: 95% CI.

**Figure 4:**
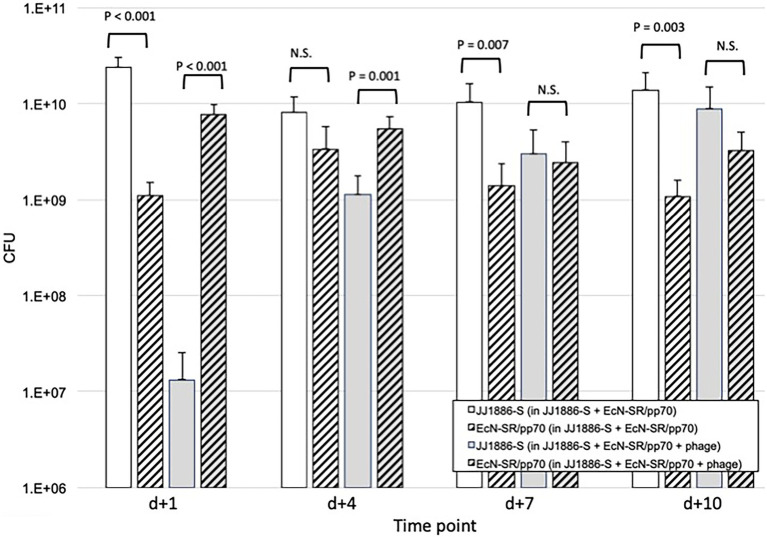
Comparative fecal counts of JJ1886-S and EcN-SR/pp70 in the murine gut colonization model. Data are group mean fecal counts of JJ1886-S (solid columns) or EcN-SR/pp70 (hashed columns; *n* = 16 mice per group) at four time points post administration (on *d*0) of both JJ1886-S and EcN-SR/pp70, with or without the five-bacteriophage cocktail (“+ phage”; gray shading). Dosing with bacteriophage and/or EcN-SR/pp70 was continued through *d* + 5 (bacteriophage) or *d* + 7 (EcN-SR/pp70). Parentheses in the key indicate which test substances the mice received. *p* values are from two-tailed paired *t*-tests. NS, not significant (*p* > 0.05). Error bars: 95% CI.

In streptomycin-fed mice, JJ1886-S, when administered by gavage on d0, established sustained gut colonization, with mean fecal counts ranging from 2.6 × 10^10^ CFU/gm (*d* + 1) to 8.3 × 10^9^ CFU/gm (*d* + 10; Figure 3). Gavage administration of bacteriophage cocktail on d0 (co-administered with JJ1886-S), *d* + 3, and *d* + 5 reduced mean fecal counts of JJ1886-S significantly on *d* + 1 (by 0.5 log_10_: *p* < 0.001); numerically but not statistically significantly on *d* + 4 and *d* + 7; and negligibly by *d* + 10. Administration of EcN-SR/pp70 by gavage on *d*0, *d* + 3, and *d* + 5, plus continuously in the drinking water, had no discernible effect on fecal counts of JJ1886-S at any time point (Figure 3), despite EcN-SR/pp70’s confirmed abundance in the gut (Figure 4).

By contrast, administration of the bacteriophage cocktail together with EcN-SR/pp70 yielded a marked synergistic effect (Figure 3). Specifically, fecal counts of JJ1886-S were reduced by 3.3 log_10_ on *d* + 1 (*p* < 0.001), 1.0 log_10_ on *d* + 4 (*p* = 0.002), and 0.5 log_10_ on *d* + 7 (*p* = 0.02). However, by *d* + 10 the JJ1886-S counts were comparable to control.

### Comparative Fecal Counts of JJ1886-S and EcN-SR/pp70 Over Time by Treatment Arm

To clarify the basis for the above findings from the gut model, additional experiments were done. Regarding the greater effect of combination therapy than of probiotic strain EcN-SR/pp70 alone, we compared over time, for mice that received EcN-SR/pp70 either alone or combined with bacteriophage cocktail, absolute fecal counts of JJ1886-S and EcN-SR/pp70, stratified by use of bacteriophage cocktail (Figure 4). Whereas in the absence of bacteriophage cocktail JJ1886-S significantly outnumbered EcN-SR/pp70 on most days (by approximately one log_10_), with the addition of bacteriophage cocktail this ratio was reversed initially, with a significant excess of EcN-SR/pp70 on *d* + 1 (by 2.8 log_10_: *p* < 0.001) and *d* + 4 (by 0.7 log_10_: *p* = 0.001), whereas by *d* + 7 the two strains were approximately equally abundant, and by *d* + 10 JJ1886-S had become numerically predominant.

### Basis for the Waning Effect of Bacteriophage Over Time

Regarding the time-limited effect of bacteriophage therapy in the gut model, we assessed several hypotheses. The possibility that bacteriophage was not being delivered to the colon, or that colonic bacteriophage lacked potency against JJ1886-S, was excluded by testing filtered extracts of fecal pellets (a surrogate for colonic contents) from *d* + 7 of bacteriophage therapy. These extracts inhibited the initial JJ1886-S, with a mean concentration reduction of 3.3 log_10_ (range, 1.0–5.5 log_10_), roughly comparable to the initial bacteriophage cocktail (mean reduction, 5.5 log_10_), whereas fecal extracts from control mice were inactive. This presumptively confirmed the presence JJ1886-active bacteriophage in the colon of bacteriophage-treated mice.

By contrast, the possibility that JJ1886-S had developed bacteriophage resistance *in vivo* during bacteriophage exposure was confirmed. Specifically, for only four of 16 bacteriophage-treated mice did the initial bacteriophage cocktail inhibit fecal isolates of JJ1886-S from *d* + 7 of bacteriophage therapy, and for one of these four mice the inhibition was only by only 0.5 log_10_. Notably, at no point did either JJ1886 or JJ1886-S exhibit evidence of acquired bacteriophage resistance after *in vitro* exposure to the bacteriophage cocktail.

### Basis for the Ineffectiveness of Probiotic Strain EcN-SR/pp70 When Used Alone

To clarify the basis for the ineffectiveness of probiotic strain EcN-SR/pp70 against colonization with JJ1886-S when used alone, despite its substantial fecal abundance (Figure 4), combinatorial experiments were done. First, to assess for possible *in vivo* development of microcin resistance by JJ1886-S during residence in the mouse gut, isolates of JJ1886-S from 32 fecal samples (four each from eight EcN-SR/pp70-treated mice, as collected on *d* + 1, *d* + 4, *d* + 7, and *d* + 10 per mouse) were tested in duplicate (*n* = 16) or triplicate (*n* = 16) for inhibition *in vitro* by supernatants from the input EcN-SR/pp70 strain. Of the eight mice, four had received and four had not received concomitant bacteriophage cocktail. Of the 32 fecal JJ1886-S isolates, 11 yielded irreproducible results (i.e., >1 log10 difference between replicate determinations, or SE ≥ 1.0), hence were uninformative, whereas the remaining 21 isolates (66%) yielded reproducible results.

The 21 isolates with reproducible results were distributed fairly evenly both by study day (4–6 isolates per day) and by concomitant administration (*n* = 9) or not (*n* = 12) of bacteriophage cocktail to the corresponding mice. Nearly all isolates (20/21, 95%) showed wild type-level inhibition by EcN-SR/pp70, with a median effect size of 5.0 log10 (range, 3.5—6.0), whereas one (from *d* + 1) showed no effect. These data largely exclude acquired microcin resistance as an explanation for either the inefficacy of probiotic strain monotherapy or the time-limited effect of combined probiotic plus bacteriophage therapy.

Second, 62 fecal isolates of EcN-SR/pp70, including 3–4 isolates each for 16 EcN-SR/pp70-treated mice (both with and without bacteriophage therapy), as selected arbitrarily from all four sampling days, were tested in triplicate for activity against the input JJ1886-S strain. Fifty isolates yielded ≥4-log_10_ inhibition and six others yielded 3.0–3.9-log_10_ inhibition. The remaining six isolates, although non-inhibitory (<1.0-log_10_ inhibition), remained ampicillin-resistant, suggesting plasmid retention. This largely excluded loss of bacteriocin production, including from plasmid segregation, by colonic-resident probiotic bacteria.

## Discussion

In this study we assessed (i) a cocktail of sewage-derived bacteriophages and (ii) an engineered microcin C7-producing probiotic *E. coli* strain, both separately and in combination, as non-antibiotic interventions against gut colonization due to *E. coli* ST131-*H*30R strains. We found that although bacteriophage therapy was extremely effective *in vivo* when assessed in the murine subcutaneous sepsis model, it was much less effective in streptomycin-fed mice for preventing gut colonization, unless combined with probiotic *E. coli*, which also was ineffective alone. In the gut model the decolonizing effect of the bacteriophage plus probiotic *E. coli* combination was especially dramatic initially and lasted at least 1 week, though it waned steadily despite ongoing administration of both agents. The findings highlight both possibilities and limitations with the studied interventions for gut decolonization, which may represent a challenging but feasible target for such interventions.

Our finding from the murine sepsis model that co-administration of a cocktail containing five *H*30R-active bacteriophages protected against sepsis due to a range of *H*30R1 and *H*30Rx strains indicates that under *in vivo* conditions the phages can target various ST131-H30R strains. This supports previous findings by others, as obtained using various rodent models of extraintestinal infection and different bacteriophages and ST131 challenge strains (Wang et al., 2006; Pouillot et al., 2012; Dufour et al., 2016; Green et al., 2017). Also, as discussed below in more detail, our bacteriophage cocktail’s marked effect in the sepsis model indicates that the same cocktail’s much lesser effect in the gut model does not reflect a general lack of *in vivo* activity but, instead, shows that the phage effect could be model or site-specific.

Our finding in the gut colonization model that the combination of bacteriophage cocktail plus probiotic *E. coli* suppressed the target *H*30Rx strain much more potently, and for longer, than did either modality alone provides clear evidence of synergy, and supports further study of such a multi-modality approach. This is in line with strategies proposed previously for addressing biofilms using bacteriophages combined with diverse adjunctive agents (Amankwah et al., 2021).

The basis for the observed synergy is unclear. Conceivably, bacteriophage treatment directed toward the target strain (JJ1886-S) gave the probiotic strain (EcN-SR/pp70) a fitness advantage over JJ1886-S. This facilitated a bloom of EcN-SR/pp70, thereby allowing it to more potently inhibit JJ1886-S, at least till JJ1886-S became bacteriophage-resistant and regained a fitness advantage over the probiotic strain. A possibly differential activity of bacteriophage vs. the probiotic strain in different intestinal microenvironments also may be relevant.

The basis for the observed inefficacy of probiotic monotherapy also is unclear. We were able to exclude both loss of microcin production by gut-resident EcN-SR/pp70 and acquisition of microcin resistance by gut-resident JJ1886-S. Possible alternative explanations include insufficient microcin expression *in vivo*; sequestration, destruction, or functional inactivation of microcin by (biotic or abiotic) components of the gut milieu, including the endogenous microbiota; insufficient mixing of gut contents to expose JJ1886-S reliably to EcN-SR/pp70; or functional microcin resistance of JJ1886-S *in vivo*, despite *in vitro* susceptibility.

The observed waning effect of bacteriophage alone, and of combined bacteriophage and probiotic *E. coli*, likely is due in part to the documented development of bacteriophage resistance, which occurred despite our use of a cocktail containing five phages, four with activity against JJ1886, and (in some mice) the probiotic strain. Based on *in vitro* testing of fecal pellets from bacteriophage-treated mice, this resistance was to the full input bacteriophage cocktail, not just its individual components, evidence that multiplicity of phages protects unreliably against resistance development. A future goal is to isolate each member of the phage cocktail from fecal samples and to test it for lytic activity against the bacteria excreted from the gut. The results of such an experiment, however, would be unlikely to contribute much toward addressing the current study’s main goal, which was to provide a conceptual proof-of-principle regarding the dual synergistic effect of the phage and probiotic mix.

The limited initial effect of bacteriophage monotherapy has multiple possible explanations, including some of those listed above for probiotic therapy. Additionally, the dosing regimen used may have been suboptimal. Exploration of higher doses and more frequent dosing is warranted. Furthermore, it is conceivable—although unlikely, given the clear appearance of their plaques—that one or more of the bacteriophages included in the present cocktail is a temperate phage, which therefore might enter the lysogenic cycle *in vivo*, thus failing to lyse the infected cell, to be amplified in the gut, and to infect and lyse additional target cells. Limiting future cocktails to confirmed lytic phages would be desirable.

The mechanism of the observed bacteriophage resistance is unknown. Conceivably, if all five bacteriophages recognize the same receptor on the target strain, a change in the expression or structure of this receptor could confer coordinate resistance to multiple bacteriophages. Regardless of its mechanism, emergence of bacteriophage resistance during bacteriophage monotherapy is a recognized “Achilles heel” of this modality (Loc-Carillo and Abedon, 2011; Lin et al., 2017; Weber-Dabrowska et al., 2017; El Haddad et al., 2019). This phenomenon may prove especially problematical with attempted gut decolonization, given the large organism burden, uncertain and probably uneven delivery of phage to all relevant compartments, presence of abundant matrix material and non-target bacteria, host digestive enzymes and antibodies, and potentially prolonged treatment courses.

With respect to gut decolonization, despite the fact that the target *H*30Rx strain was never fully eliminated (whereas full elimination would be clinically desirable) and by *d* + 10 no intervention had any discernible effect, this study could be regarded as providing proof of concept that the combination of bacteriophage plus a probiotic may be able to diminish the gut population of MDROs, and specifically ST131-*H*30R, which is an especially effective gut colonizer (Tchesnokova et al., 2020; Johnson et al., 2022). However, considerable work would be needed to translate this concept into an effective therapeutic modality and to address ethical and practical concerns related to genetically modified organisms.

Study limitations include that the bacteriophages were characterized only by assessing their spectrum and strength of lytic activity against a broad range of *E. coli* strains; sequencing them to define their identity and lytic vs. temperate nature, and imaging them by transmission electron microscopy to define their morphology and homogeneity is a future goal. Additionally, phage resistance mechanisms were not characterized; the mechanistic basis for the observed phage-probiotic synergy was not defined; the model systems used, which are highly artificial and idealized, relied on a single dosing regimen; and the bacteriophage preparations may have contained residual endotoxin, which would need to be removed before clinical use. Closing these gaps is another future goal. Strengths include the use of complementary interventions and animal models, assessment of multiple clinical isolates from the epidemiologically important *H*30R subclone, and preliminary exploration of the basis for the gut model’s unexpected findings.

In summary, we found that in mice a novel bacteriophage cocktail was only marginally effective against gut colonization when used alone, but was significantly more effective when combined with a probiotic *E. coli* strain, which by contrast was completely ineffective alone. Emergence of bacteriophage resistance during attempted gut decolonization may have limited the durability of effect. These findings provide proof of concept for combined bacteriophage-plus-probiotic therapy to address gut colonization with MDROs and suggest that, despite eventual emergence of bacteriophage resistance in ST131-*H*30Rx, gut colonization may be a feasible, albeit challenging, target whereby non-antibiotic measures could reduce the burden of MDROs.

## Data Availability Statement

The raw data supporting the conclusions of this article will be made available by the authors, without undue reservation.

## Ethics Statement

The animal study was reviewed and approved by Institutional Animal Care and Use Committee, Minneapolis VA Health Care System.

## Author Contributions

SP: data acquisition, data analysis, writing, revising, and preparing the figures. BJ: provision of reagents, data acquisition, writing, and revising. DK: provision of reagents, data acquisition, and revising. CC: provision of reagents and revising. ES: site supervision, funding, concept, and revising. JJ: concept, funding, overall supervision, preparing the figures, writing, and revising. All authors contributed to the article and approved the submitted version.

## Funding

This work was supported in part by the Office of Research Development, Department of Veterans Affairs, and by NIH grants R21AI147575 and R01AI106007 (to ES).

## Conflict of Interest

The authors declare that the research was conducted in the absence of any commercial or financial relationships that could be construed as a potential conflict of interest.

## Publisher’s Note

All claims expressed in this article are solely those of the authors and do not necessarily represent those of their affiliated organizations, or those of the publisher, the editors and the reviewers. Any product that may be evaluated in this article, or claim that may be made by its manufacturer, is not guaranteed or endorsed by the publisher.
